# Integrated Bioinformatics Analysis Identifies ELAVL1 and APP as Candidate Crucial Genes for Crohn's Disease

**DOI:** 10.1155/2020/3067273

**Published:** 2020-07-17

**Authors:** Heli Li, Qianru Li, Shiran Sun, Ping Lei, Xiong Cai, Guanxin Shen

**Affiliations:** ^1^Department of Immunology, School of Basic Medicine, Tongji Medical College, Huazhong University of Science and Technology, Wuhan, China 430030; ^2^Department of Hepatobiliary Surgery, Union Hospital, Tongji Medical College, Huazhong University of Science and Technology, Wuhan 430022, China; ^3^Department of Dermatology, Union Hospital, Tongji Medical College, Huazhong University of Science and Technology, Wuhan 430022, China

## Abstract

Immune imbalance and barrier destruction of intestinal mucosa are the central pathogenic factors of Crohn's disease (CD). In this study, three independent microarray studies of CD were integrated and 9912 differentially expressed genes (DEGs) were analysed by NetworkAnalyst to screen candidate crucial genes. NetworkAnalyst identified ELAV-like RNA binding protein 1 (ELAVL1) as the most crucial upregulated gene and amyloid-*β* precursor protein (APP) as the most crucial downregulated gene in peripheral blood of CD patients. By computing significance with hypergeometric test based on the KEGG pathway database, upregulated DEGs highlight the pathways of T cell receptor signaling and the differentiation of T helpers. Downregulated DEGs were found enriched in pathways in multiple cancers, MAPK signaling, Rap1 signaling, and PI3K-AKT signaling. Further taking all DEGs together, Gene Set Enrichment Analysis (GSEA) brought out the NOD-like receptor (NLR) signaling pathway which could be regulated by ELAVL1. xCell found decreased naïve and differentiated T cell proportions in the peripheral blood of CD patients suggesting T cell migration to the intestinal tissue and/or exhaustion. Further, ELAVL1 expression correlating with multiple T cell proportions suggests that ELAVL1 may regulate T cell activation. These findings illustrated that ELAVL1 and APP were candidate crucial genes in the peripheral blood of CD patients. ELAVL1 possibly acts as a key regulator of T cell activation via the NLR signaling pathway. APP might be a downstream effector of infliximab treatment connecting with MAPK signaling.

## 1. Introduction

Crohn's disease (CD), as a systemic inflammatory disease, mainly influences the gastrointestinal tract with a wide range of contributing factors including host genetics, immune system, environmental exposures, and the gut microbiome [1]. Although the pathogenesis is complex, the decades of studies have illustrated that CD is caused by environmental factors that broke the mucosal barrier and increased the luminal antigens into the lamina propria [2]. Different innate immune cells, like dendritic cells, recognize the antigens via pattern recognition receptors, such as Toll-like receptors (TLR) and NLR, and then regulate the activation of T cells [3].

Among the factors associated with CD in immune system, T cells were highlighted in CD pathology because about 200 CD risk loci are involved in T cell signaling [4, 5]. Furthermore, the CD4^+^ T cell, including Th1, Th17, and regulatory T (Treg) cells, were associated with the severity of CD, particularly with active inflammation [2]. In addition, CD8^+^ T cell transcriptional signatures were identified as reliable prognostic biomarkers in the blood of CD patients [6, 7]. Likewise, numerous studies are devoted to the development of diagnostic methods related to T cells for CD. For example, four CD-related differentially methylated regions were identified in the whole blood [8]. In addition, one T cell subtype from the blood of CD patients is enriched for the CD-risk gene [9]. Nevertheless, the key regulators and mechanisms regulating T cell activation in the blood of CD have not been fully described. Thus, we integrated three independent microarray studies to screen crucial genes in the blood of CD patients.

We performed an integrated bioinformatics analysis to find key regulators by NetworkAnalyst [10], a web-based visual analytics platform. Indeed, the utility of NetworkAnalyst to identify DEGs and the pathway has been demonstrated recently. For example, network analyses identified HNF4A and PTBP1 as effective biomarkers for Parkinson's disease [11] and 873 DEGs genetically related to insulin resistance [12].

In this study, we launched a network-based bioinformatics analysis in an attempt to screen DEGs in the blood of CD patients, followed by the KEGG pathway enrichment analysis, GSEA, and interaction network of DEGs. We identified ELAVL1 and APP, previously implicated in regulating the activation of innate immunity [13, 14] and T cells [15], the most significant up- and downregulated genes. Furthermore, GSEA data show that NOD-like receptor signaling-associated gene signatures are enriched and the xCell analysis indicated that most T cells are significantly decreased in the three microarray studies, which could help in understanding the role of ELAVL1 and APP in the blood of CD patients.

## 2. Materials and Methods

### 2.1. Microarray Source

The microarray studies of CD were downloaded from the Gene Expression Omnibus (GEO) by using the terms “Crohn's disease” and “blood”. Three microarray studies were selected for subsequent integrative analysis. GSE86434 included 23 newly diagnosed CD patients and 24 healthy controls (HC). GSE94648 included 22 healthy controls, 9 inactive CD patients and 41 active CD patients. GSE119600 included 47 adult healthy controls, 48 adult CD patients, and 47 child CD patients. PRJEB28822 was chosen to be the verified datasets from the European Nucleotide Archive (ENA), which included 102 pediatric IBD patients and 51 controls, and 95 adult IBD patients and 46 controls, separately.

### 2.2. Gene Expression and Connectivity Correlation Assay

To assess the comparability of two datasets, measures of average gene expression and overall connectivity between two datasets were correlated. The higher the correlations, the better the chance of finding similarities between the two datasets at subsequent stages of the analysis. All enrolled datasets with or without batch effect adjustment were examined for average expression correlation (correlation between expression ranks of genes in two studies) and connectivity correlation (correlation between connectivity ranks of genes in two studies) by R package WGCNA [16]. Better comparable datasets were indicated by greater positive correlations and more significant *p* values.

### 2.3. Integrated Network-Based Bioinformatics Analysis

We performed integrated bioinformatics analysis using NetworkAnalyst [10] in accordance with the protocol [17]. After annotating the gene probes to a common Entrez ID, these datasets were normalized per platform requirement and uploaded to the website. DEGs of each dataset were defined by *p* < 0.05 and log2 fold change > 1. The Venn diagram of DEGs was generated by the FunRich tool (version 3.1).

Network-based bioinformatics analysis was performed by NetworkAnalyst according to the pipeline described. The whole blood-specific protein-protein interaction (PPI) of the top 20 DEGs and up- or downregulated genes was constructed per protocol [17]; then, the KEGG pathway enrichment analysis was performed by applying hypergeometric test.

### 2.4. Gene Set Enrichment Analysis

Preranked GSEA was analysed using a NetworkAnalyst module powered by R package fgsea [10]. All DEGs ranked by fold changes were put into the analysis, and the results was visualized as interactive heatmaps.

### 2.5. In Silico Immune Cell Type Enrichment Analysis

xCell, a gene signature-based method reliably portraying the cellular heterogeneity landscape of tissue expression profiles, was performed to explore immune cell types [18], and the proportion of T cells were obtained for each CD and HC sample per instruction.

### 2.6. Statistical Analysis

All basic statistical analyses, including the Mann–Whitney test, Pearson correlation, and Spearman correlation were calculated by R software. A *p* value < 0.05 was considered to be statistically significant. Data were represented by mean and standard deviation (SD) or median and quantile depending on distributions.

## 3. Results

### 3.1. Screening DEGs by Integrated Bioinformatics Analysis

**Figure 1:**
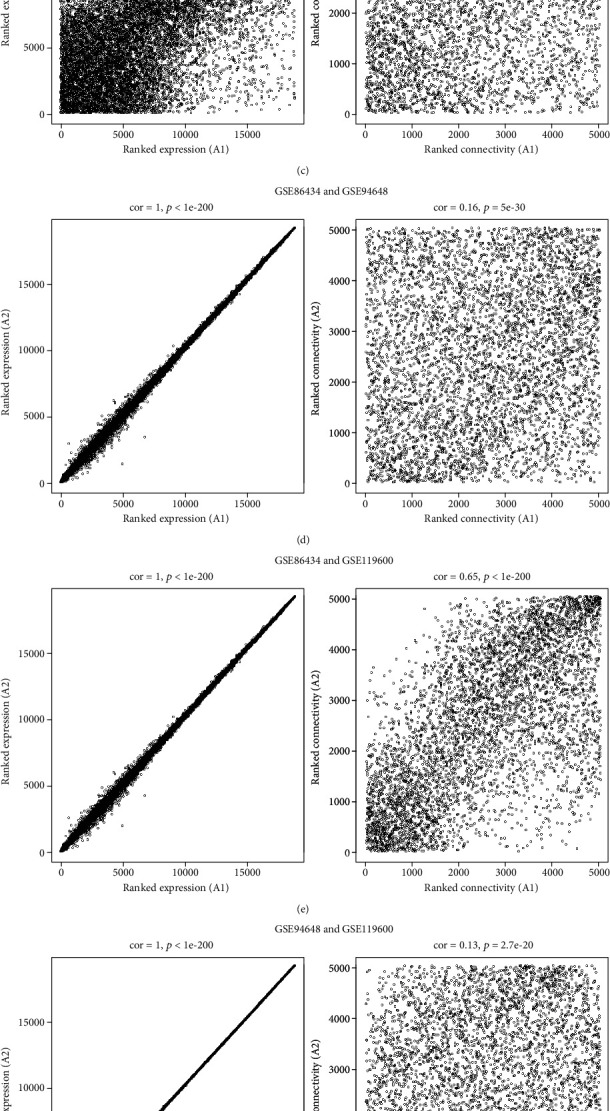
Screening DEGs by integrated bioinformatics analysis. The gene expression and connectivity of GSE86434 and GSE94648 (a), GSE86434 and GSE119600 (b), and GSE94648 and GSE119600 (c). After moving batch effects with parametric empirical Bayes frameworks provided by the ComBat function in R package sva, the gene expression and connectivity of GSE86434 and GSE94648 (d), GSE86434 and GSE119600 (e), and GSE94648 and GSE119600 (f). (g) PCA plot for sample clustering of all datasets without batch effect adjustment (A) and with batch effect adjustment (B). (h) Venn diagram of integrative analysis DEGs (Integrated-DE) and DEGs from each individual dataset (Individual-DE).

Three microarray studies (Table 1) were analysed using NetworkAnalyst to screen DEGs in the blood of CD patients. Firstly, these datasets were preprocessed to ensure they are comparable enough. The correlations were positive and the *p* values were significant in all cases before batch effect adjustments (Figures 1(a)–1(c)). The correlations (cor) for the gene expression and connectivity of GSE86434 and GSE119600 (expression, cor = 0.97, *p* < 1*e* − 200; connectivity, cor = 0.74, *p* < 1*e* − 200) were better than GSE86434 and GSE94648 (expression, cor = 0.76, *p* < 1*e* − 200; connectivity, cor = 0.34, *p* < 1.6e − 135) or GSE94648 and GSE119600 (expression, cor = 0.75, *p* < 1*e* − 200; connectivity, cor = 0.34, *p* < 1.6*e* − 135). GSE86434 and GSE119600 were from the same platform (Illumina HumanHT-12 V4.0 expression bead chip). Thus, it is consistent with the notion that datasets from the same platform are more comparable than datasets from different platforms. To remove this batch effect, parametric empirical Bayes frameworks provided by ComBat function were applied by NetworkAnalyst. And the results after batch effect moving were shown in Figures 1(d)–1(f), indicating that the processed data are more comparable. PCA plots with or without batch effect adjustment were visualized in Figure 1(g).

Integrated bioinformatics analysis identified 9912 DEGs in the three microarray studies, in which 4705 genes were upregulated and 5207 were downregulated in CD compared with HC. A Venn diagram of integrative analysis DEGs and individual DEGs are shown in Figure 1(h). There were 1361 genes specifically classified by integrated bioinformatics analysis that are weakly but consistently expressed among the three datasets. 2511 genes were identified as lost genes, which are expressed in individual datasets but not in the integrated datasets.

### 3.2. Top 20 Hub Genes Are Identified by Network-Based Analysis

**Figure 2:**
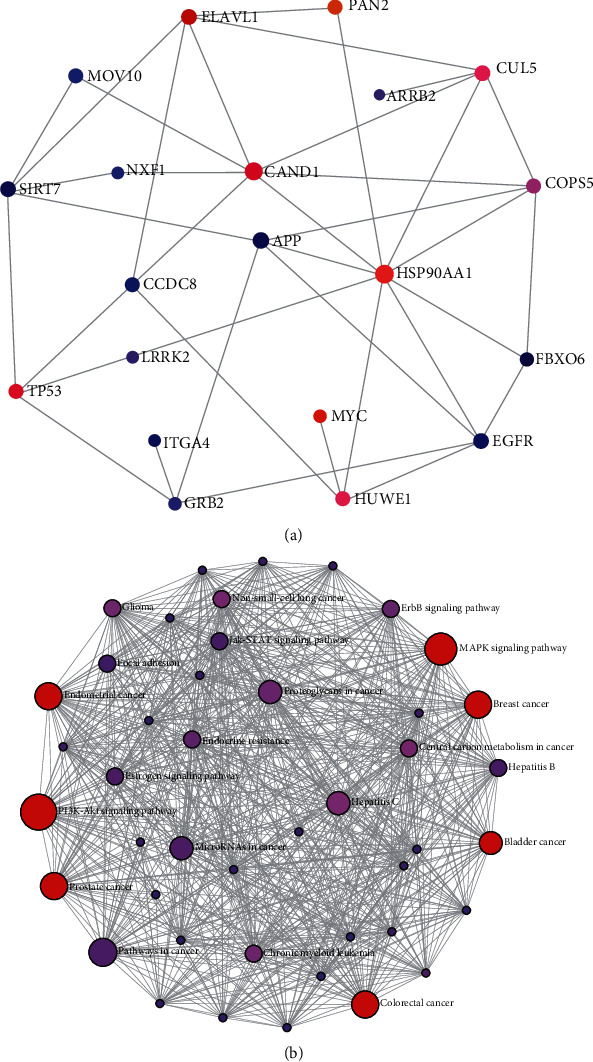
Top 20 hub genes are identified by network-based analysis. (a) Zero-order interaction network and (b) enrichment network of top 20 hub genes.

Firstly, we put all DEGs into network-based analysis per instruction [10]. According to the degree of centrality (DC) and betweenness (BC), the most highly ranked node was APP (DC = 1056; BC = 4730653.29) followed by EGFR (DC = 580; BC = 1711464.65) and ELAVL1 (DC = 574; BC = 1999915.6) and the top 20 hub genes were presented (Table 2). The results of the KEGG pathway enrichment analysis of these 20 genes were shown in Table 3. The whole blood-specific PPI (Figure 2(a)) and enrichment network (Figure 2(b)) were generated by NetworkAnalyst. The zero-order interaction network of these genes contained 20 nodes and 38 edges (Figure 2(a)). The results of pathway enrichment indicated that variables in the blood transcriptome of CD were closely connected to several cancers including colorectal cancer, and also with the PI3K-AKT and MAPK signaling pathways.

### 3.3. ELAVL1 and APP Are Hub Genes Measured by Network Analysis

**Figure 3:**
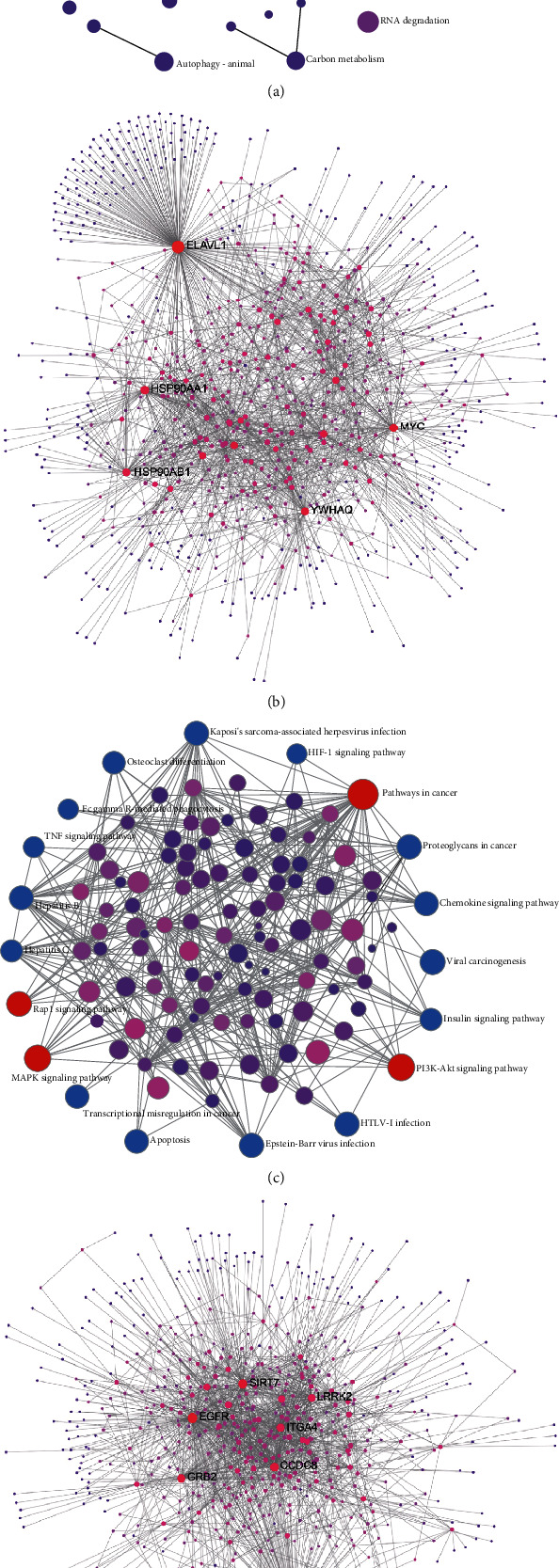
ELAVL1 and APP are hub genes measured by network analysis. (a) Enrichment network and (b) zero-order interaction network of upregulated genes identified by network-based analysis. (c) Enrichment network and (d) zero-order interaction network of downregulated genes identified by network-based analysis.

Then, we analysed pathway enriched in up- or downregulated DEGs separately to explore key regulators in the blood of CD patients, by NetworkAnalyst [10]. Upregulated genes in the blood of CD were enriched in the pathways, including T cell receptor signaling pathway, primary immunodeficiency, Th1 and Th2 cell differentiation, Th17 cell differentiation, and NF-kappa B signaling pathway (Figure 3(a)). The most crucial gene among upregulated DEGs was ELAVL1 (DC = 357; BC = 709523.17) (Figure 3(b)). Downregulated genes in the blood of CD were enriched in the pathways, including pathways in cancer, MAPK signaling pathway, Rap1 signaling pathway, and PI3K-AKT signaling pathway (Figure 3(c)). The most crucial gene among downregulated DEGs was APP (BC = 1244517.75; DC = 492) (Figure 3(d)).

Furthermore, we analysed an AmpliSeq study (PRJEB28822) in the whole peripheral blood of IBD patients [21]. APP expression was significantly reduced in pediatric IBD patients compared with HC (FC = −0.54, *p* < 0.001), while ELAVL1 was significantly elevated (FC = 0.15, *p* < 0.05).

### 3.4. NOD-like Receptor Signaling Pathway Is Enriched in the Blood of CD by GSEA Analysis

**Figure 4:**
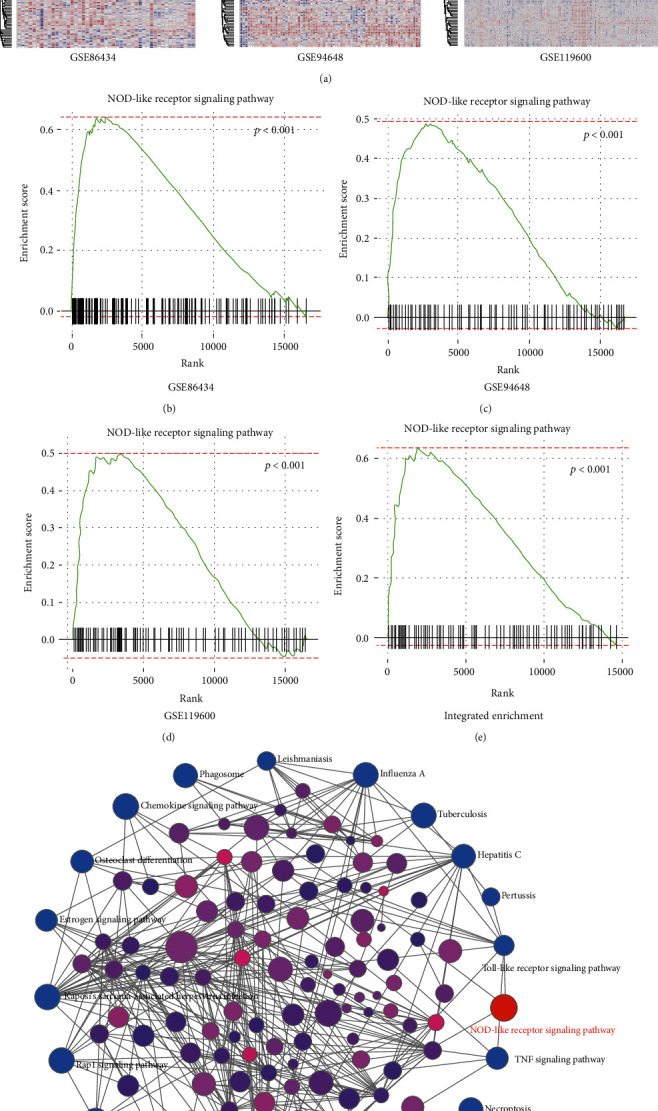
NOD-like receptor signaling pathway is enriched in the blood of CD by GSE analysis. (a) GSEA-generated heatmaps of core enrichment genes in the NOD-like receptor signaling pathway upregulated in CD patients of three datasets. (b–d) Enrichment maps were used for the visualization of NOD-like receptor signaling pathway enrichment results separately. (e) Integrated enrichment network of the three microarray datasets was generated by NetworkAnalyst. (f) Enrichment map visualizes NOD-like receptor signaling pathway enrichment results from integrated data.

To further clarify the possible mechanism of CD, preranked GSEA was performed to analyse the three microarray datasets separately or integrally selecting fold change as the gene ranking methods (Table 4). As shown in Table 4 and Figures 4(a)–4(d), GSEA analysis suggested that NOD-like receptor signaling pathway is the only enriched gene set we can find in the top 10 enriched gene sets of all three datasets. In addition, the integrated dataset analysed by GSEA also indicated that NOD-like receptor signaling pathway was enriched in CD patients (Figures 4(e) and 4(f)). These results revealed that NOD-like receptor signaling may act as a crucial role in the development of CD.

### 3.5. ELAVL1 Expression Correlates with Genes in NLR Signaling Pathway, and APP Expression Is Associated with MAPK Signaling Cross Multiple Datasets

**Figure 5:**
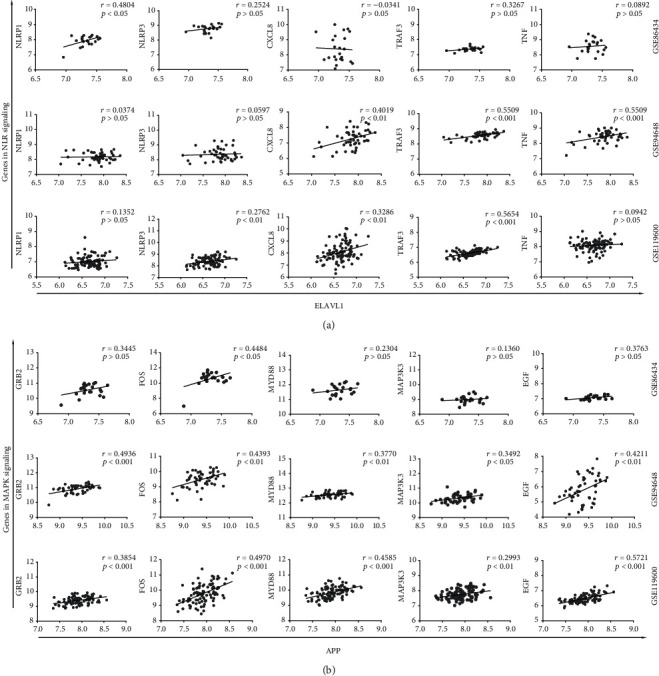
The transcriptome association between candidate crucial genes and top enriched signaling pathways in the peripheral blood of CD patients. The gene expression correlation between ELAVL1 and selected genes in NLR signaling (a) and correlation between APP and selected genes in MAPK signaling (b) in CD patients analysed using the expression data from the three datasets were represented by scatterplots with regression lines. Pearson correlation coefficients (*r*) and *p* values were calculated and shown.

To further explore and validate the association between candidate crucial genes and top enriched signaling pathways in CD patients, we analysed the direct correlation between ELAVL1 and major genes in the NLR pathway and correlation between APP and major genes in MAPK signaling by the Pearson correlation. As shown in Figure 5(a), genes in the NLR signaling pathway, including NLRP1, NLRP3, CXCL8, TRAK4, and TNF, were correlated with the expression of ELAVL1 at least in one of the datasets. Additionally, GRB2, FOS, MYD88, MAP3K3, and EGF were found associated with the expression of APP (Figure 5(b)). These results strongly supported the direct correlation between ELAVL1 and NLR signaling, as well as correlations between APP and MAPK signaling in the peripheral blood of CD patients. A slight inconsistency of correlations and significances among three datasets may be due to varied sample sizes and markable heterogeneity of the disease.

### 3.6. Naïve and Differentiated T Cell Proportions in the Peripheral Blood of CD Patients Are Decreased Compared to Health Controls

**Figure 6:**
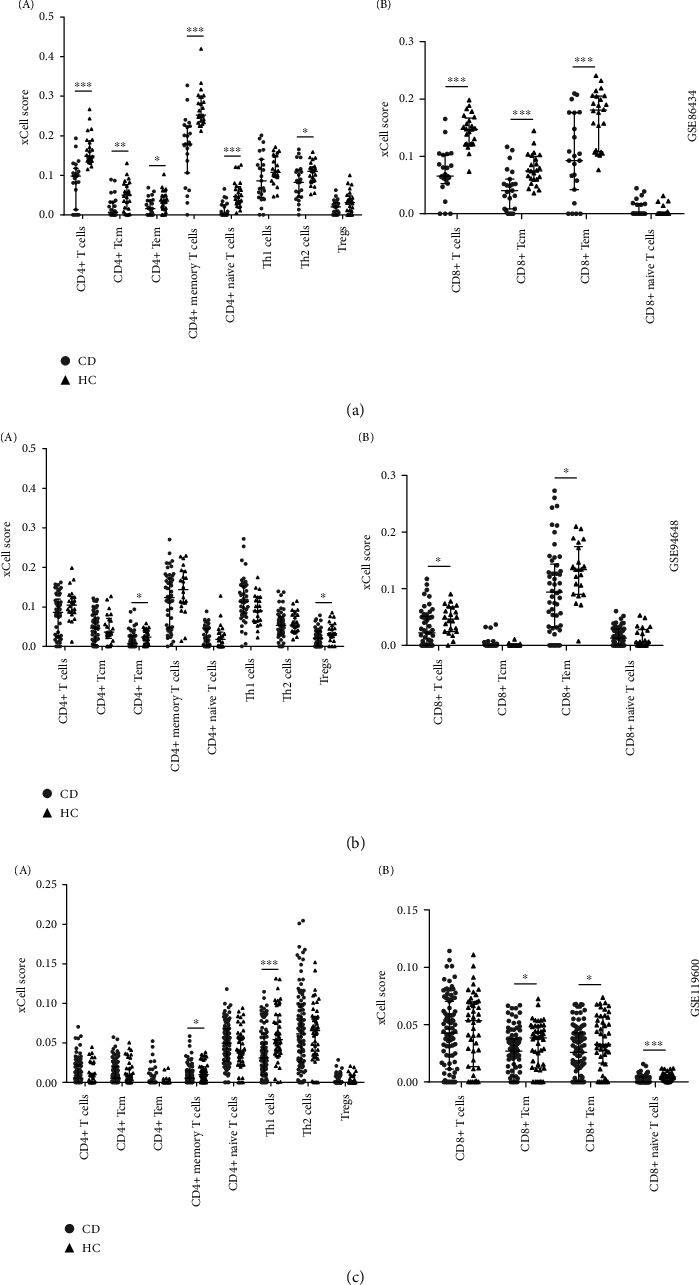
T cell proportions are significantly altered in the peripheral blood of CD comparing to health control. The scatterplots show the xCell scores for CD4^+^ (A) and CD8^+^ (B) T cells of CD and HC from GSE86434 (a), GSE94648 (b) and GSE119600 (c). ^∗^*p* < 0.05, ^∗∗^*p* < 0.01, and ^∗∗∗^*p* < 0.001.

Previous studies have indicated that distinct T cell subsets, such as Th1/Th17, Tregs, memory, and naïve T cells, are activated in the gut of CD patients. Therefore, we performed xCell to explore the signature of T cells in the peripheral blood of CD from the three datasets and found that the altered T cell proportions were one of the hallmarks in the peripheral blood of CD (Figure 6). Specifically, CD4^+^ T cells were obviously decreased in GSE86434 (Figure 6(a)) and GSE94648 (Figure 6(b); similar trends were also shown in GSE119600 (Figure 6(c)). Because of the essential function of CD4^+^ T cells in helping the activation of CD8^+^ T cells, these data also show the decrease of CD8^+^ T cells in GSE86434 (Figure 6(a)) and GSE94648 (Figure 6(b)), as well as in GSE119600 (Figure 6(b). Similarly, the central memory CD8^+^ T (Tcm) cells were clearly decreased in all three datasets. Of note, the effector memory CD8^+^ T (Tem) cells were apparently decreased in the three datasets, which may be a new hallmark in the blood of CD patients. In addition, Th1 cells, the cell type mostly relevant to CD, were disordered in the three datasets. Since T cells are activated and recruited to the intestinal tissue in CD to resist bacterial infections and continuously activated T cells can be exhausted, most T cell subsets decreased in the peripheral blood of CD can be interpreted by T cell migration to the intestinal tissue and/or exhaustion.

### 3.7. ELAVL1 Expression Correlates with Various T Cell Subset Proportions in the Peripheral Blood of CD Patients

**Figure 7:**
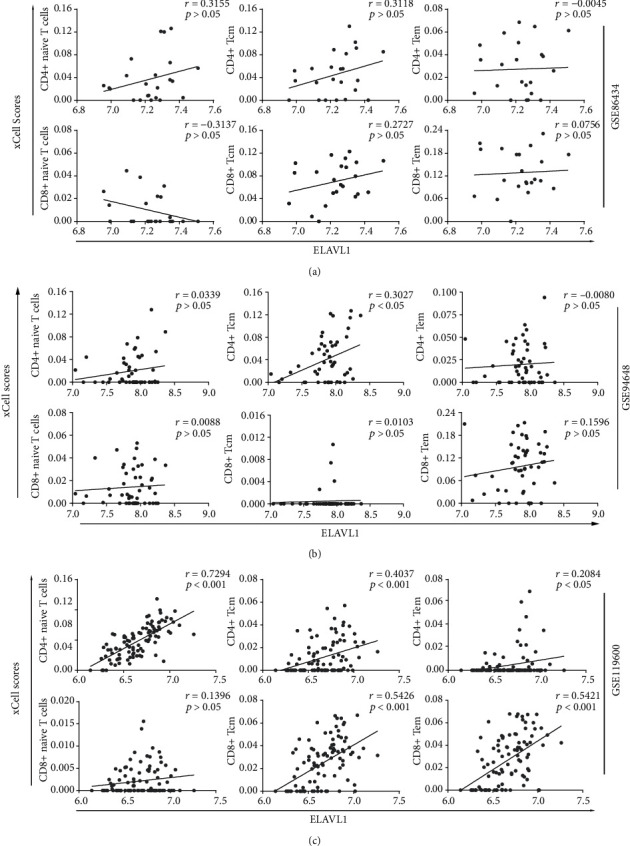
ELAVL1 expression correlates with multiple T cell subset activation in the peripheral blood of CD patients. The association between ELAVL1 and the xCell scores of selected T cell subsets of CD patients were analysed using the expression data from GSE86434 (a), GSE94648 (b), and GSE119600 (c). Scatterplots were represented with regression lines. The Spearman correlation coefficients (*r*) and *p* values were calculated and shown.

Since we have found that ELAVL1 is closely associated to NLR signaling, which has an important impact on priming the activation of T cells, we further investigate the association between ELAVL1 expression and the xCell scores of T cells in CD patients by the Spearman correlation. As shown in Figures 7(a)–7(c), different T cell subsets, including CD4^+^ naive T cells, CD4^+^ Tcm, CD4^+^ Tem, CD8^+^ naive T cells, CD8^+^ Tcm, and CD8^+^ Tem, were found positively correlated with ELAVL1 expression in dataset GSE119600 including 95 CD patients (Figure 7(c)). These results suggest that the upregulated expression of ELAVL1 is associated with T cell activation, probably through promoting the NLR signaling pathway. While in GSE86434 and GSE94648, the absence of a significant correlation may due to limited CD sample sizes of the two datasets.

## 4. Discussion

Up to date, the immunological pathogenesis of CD continues to be complex, and finding the key regulators of the immune response is essential for knowing the disease and improving the clinical management of CD. Specifically, investigations focusing on exploring CD pathogenesis and identifying therapeutic targets are warranted for the discovery of effective drugs. Here, we identified the candidate crucial genes in the peripheral blood of CD by a network-based analysis of NCBI GEO datasets GSE86434, GSE94648, and GSE119600. Integrative analysis identified 9912 DEGs among these datasets. ELAVL1 and APP were confirmed as the most significant up- and downregulated genes by NetworkAnalyst. Furthermore, the differential expression significances of the two hub genes were validated by an AmpliSeq dataset PRJEB28822, indicating that ELAVL1 and APP may be potential biomarkers for CD patients.

ELAVL1, also known as Hu antigen R (HuR), is an abundant RNA binding protein that can affect the stability and translation of many RNAs and participate in the regulation of chronic inflammation and cancer progression [22, 23]. It has been identified that ELAVL1 was increased after cellular stress with protective activities [24], which is positively regulated by NF-*κ*B and Smads [25]. Furthermore, the increase of ELAVL1 was confirmed to suppress inflammatory responses in mice [23], suggesting the important role of ELAVL1 as a posttranscriptional mediator for inflammation. However, it is worth noting that the increased expression of ELAVL1 is identified to promote the overexpression of COX-2, and thus contributing to the growth of colon cancer, whose risk is increased in the setting of CD [26, 27]. Moreover, the influence of ELAVL1 in promoting malignant transformation has been well documented in multiple cancers [28–32]. That is in consistent with our pathway enrichment of top 20 hub genes indicating that variables in blood transcriptome of CD were closely connected to several cancers including colorectal cancer. The pathway enrichment of top 20 hub genes also shed light on the PI3K-AKT and MAPK signaling pathways, that is in line with investigations that phosphorylation by p38 MAPK results in the accumulation of ELAVL1 in the cytoplasm [33] and the elevation of ELAVL1 is essential for enhancing the proliferation of gastric cancer cells, which depends on the activation of PI3K-AKT and NF-*κ*B signaling [34]. Our results highlight the crucial role of ELAVL1 in connecting inflammatory meditation with tumorigenesis, suggesting the potential role of ELAVL1 in carcinogenesis of colorectal cancer in the background of CD.

APP has been known as central to the pathogenesis of Alzheimer's disease (AD) and has been confirmed as the potential biomarker in predicting brain amyloid-*β* burden [35]. Although the specific mechanism coexisting in AD and CD is still unclear, it has been demonstrated that there are genetic factors overlapping between AD and CD [36]. Besides, the immune response is considered one of major contributors in both AD and CD. A recent review highlights the influence of the peripheral immune system in AD [37], such as monocytes and lymphocytes, which have been linked to CD in a number of studies [38]. This overlap in the pathogenesis between the two diseases may suggest the potential central role of APP in CD pathogenesis. Though literature about APP in CD is very limited, we can still note that Apolipoprotein E (APOE), widely found increased in serum of CD patients who were primary nonrespondents or had responded clinically and serologically after infliximab treatment of CD [39], can increase transcription of APP significantly [40]. Next, APP can active the MAPK signaling pathway [41, 42], which is one of the top enriched gene sets in our pathway enrichment analysis of top 20 hub genes. Since detailed mechanism about infliximab treatment in CD has not been fully elucidated, APP may be predicted as a downstream effector of the treatment which is worthy of further investigation.

The results from GSEA of the individual and overall databases highlight the NOD-like receptor signaling pathway in the blood of CD patients. NOD1 and NOD2, members of the NOD-like receptor family, are two important mediators of inflammation induced by endoplasmic reticulum stress, which is a major contributor to CD [43, 44]. In addition to the roles in regulating innate immune responses, there are plenty of evidences that NOD1 and NOD2 signaling have an impact on adaptive immune responses. In mice, NOD1 and NOD2 signaling are involved in the activation of Th1, Th2, and Th17 cells [45]. NOD2 also can drive CD8^+^ T cell activation via the cross-presentation pathway [46]. Our integrative analysis also highlights the pathway of the T cell receptor signaling pathway and the differentiation of Th1, Th2, and Th17, which act essential roles in the pathogenesis of CD [38, 47, 48]. It is reasonable for us to assume that the NLR signaling pathway may work as a key regulator of CD pathology by activating T cells. Moreover, the highest ranking of the integrated dataset analysed by GSEA is the MAPK signaling pathway, followed by the Rap1 signaling pathway. As known, Rap1 plays an essential role in regulating the activation of MAPK [49], which is definitely involved in the development of CD [50] and is an effective target for the treatment of CD [51]. Therefore, it will be interesting to focus linkage between APP and MAPK and investigation of their role in the progression of CD seems to be promising.

The adaptive immune system is considered the key regulator of the pathogenesis of CD [3]. When the intestinal barrier is broken, pattern recognition receptors, such as TLR [52] and NLR [53], recognize the microbe-associated molecular patterns, thus promoting the activation and differentiation of T cells [54]. Especially, CD4^+^ T cells are activated and differentiated into Th1/Th17, help the development of memory T cells, and then recruited to the gut to fight against the infection of bacteria, fungi, and viruses [55]. However, continuous T cell activation will aggravate inflammatory damage [2] and will even lead to the exhaustion of T cells [56–58]. Of note, the exhaustion of T cells regularly impedes the ability to defeat viral infection [59] and promote the occurrence of cancer [60]. Interestingly, the data of xCell indicated that CD4^+^ and CD8^+^ T cell proportions are obviously reduced in the blood, which may be caused by the increased migration of T cells into the gut and/or the exhaustion of T cells. Consistent with FACS data from other literatures [61–63], our xCell analysis highlight the decrease of CD8^+^ Tem as a general signature in the blood of CD, which is conventionally considered a cytotoxic cell type to defect the infection of virus. Although little is known about the detail mechanism, these phenomena suggest that the decrease of T cells, especially the CD8^+^ Tem, in the peripheral blood may be a novel feature for certain CD patients.

As known, ELAVL1/HuR acts as a central posttranscriptional regulator of NOD2 expression, and HuR silencing can reduce NOD2 expression and mRNA stability [64]. In addition, HuR stimulated by integrin engagement and the level of HuR nuclear export are definitely involved in the activation of T cells [65]. Interestingly, we found that ELAVL1 expression showed strong positive correlations with multiple T cell subset proportions in GSE119600. The association supports the idea that ELAVL1 can modulate the immune response by activating the T cells. However, we cannot demonstrate the regulatory roles of ELAVL1 on T cell activation in the current transcriptome analysis; further investigation in an animal model will be meaningful and straightforward.

**Figure 8:**
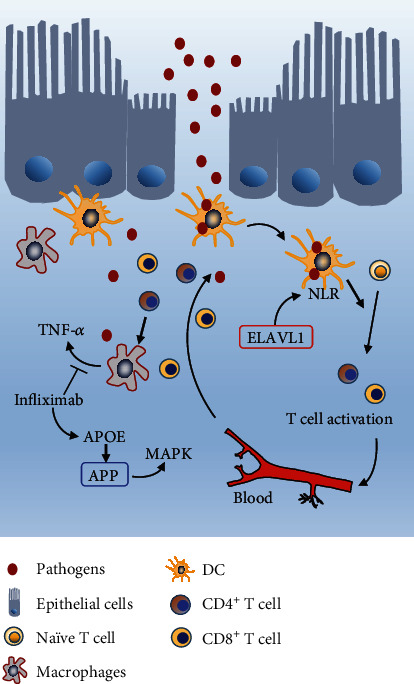
The schematic diagram illustrating ELAVL1/NLR, APP/MAPK signaling, and T cell migration and/or exhaustion in CD pathogenesis.

In conclusion, our study suggests that ELAVL1 and APP are candidate crucial genes in the blood of CD and highlights the function of the NLR signaling pathway in priming the activation of T cells of CD. ELAVL1 may modulate the immune response of CD via the NLR signaling pathway and in turn regulate T cells status. APP could be a downstream effector of infliximab treatment connecting with MAPK signaling (a schematic diagram representing a possible mechanism is shown in Figure 8). Our analysis will be helpful for further investigation and understanding of the mechanism of ELAVL1 and APP in CD pathogenesis.

## Figures and Tables

**Table 1:** Gene expression datasets used in this study.

Disease	Datasets	Platform	Cases	Controls	References
Exploration					
CD	GSE86434	GPL10558	23	24	[8]
CD	GSE94648	GPL19109	50	22	[19]
CD	GSE119600	GPL10558	95	47	[20]
Validation					
IBD	PRJEB28822	Ion AmpliSeq Transcriptome Human Gene Expression Panel	102	51	[21]
			Pediatric	Pediatric	
			95	46	
			Adult	Adult	

**Table 2:** Top 20 hub genes with DC and BC.

Gene	DC	BC	Expression
APP	1056	4730653	-33.853
EGFR	580	1711465	-15.619
ELAVL1	574	1999916	34.987
CAND1	381	578227.8	24.477
ITGA4	370	457397.4	-15.926
SIRT7	357	744489.5	-45.873
FBXO6	352	760217.7	-53.7
CCDC8	322	504043	-15.191
GRB2	303	630311.8	-26.82
TP53	281	621198.7	20.389
MOV10	260	573433.5	-20.408
NXF1	241	496040.6	-23.619
MYC	239	458057	21.06
HSP90AA1	232	486480.7	28.182
HUWE1	232	242665.7	26.081
LRRK2	223	216252.1	-31.578
COPS5	220	313401.8	48.805
ARRB2	219	230981.9	-42.185
PAN2	217	168810.6	30.93
CUL5	208	174910.9	22.703

**Table 3:** KEGG analysis of the 20 hub genes identified by network-based analysis.

Term	Count	Gene	*p* value
Endometrial cancer	4	20%	3.65*e*-6
Colorectal cancer	4	20%	1.77*e*-5
Prostate cancer	4	20%	2.85*e*-5
PI3K-Akt signaling pathway	6	30%	3.1*e*-5
Bladder cancer	3	15%	6.01*e*-5
Breast cancer	4	20%	1.45*e*-4
MAPK signaling pathway	5	25%	1.71*e*-4

**Table 4:** GSEA analysis of the three microarray datasets.

GSE86434		GSE94648		GSE119600		Integrated	
Name	Enrichment score	Name	Enrichment score	Name	Enrichment score	Name	Enrichment score
NOD-like receptor signaling pathway	0.64066	Huntington's disease	0.453565	Pathways in cancer	0.404973	MAPK signaling pathway	0.446547
Kaposi's sarcoma-associated herpesvirus infection	0.473815	Alzheimer's disease	0.486581	Endocytosis	0.426882	Kaposi's sarcoma-associated herpesvirus infection	0.499763
Tuberculosis	0.48882	Alcoholism	0.509264	MAPK signaling pathway	0.462268	Rap1 signaling pathway	0.517768
Chemokine signaling pathway	0.474491	NOD-like receptor signaling pathway	0.492519	Proteoglycans in cancer	0.46461	NOD-like receptor signaling pathway	0.633331
Influenza A	0.504921	Nonalcoholic fatty liver disease (NAFLD)	0.487171	Kaposi's sarcoma-associated herpesvirus infection	0.454893	Tuberculosis	0.549604
Alcoholism	0.597721	Parkinson's disease	0.573813	Regulation of actin cytoskeleton	0.48289	Influenza A	0.505989
Necroptosis	0.526145	Lysosome	0.453183	Rap1 signaling pathway	0.524153	Hepatitis B	0.536561
Phagosome	0.515787	Oxidative phosphorylation	0.614682	Tuberculosis	0.47918	Alcoholism	0.488690
Hepatitis C	0.558024	Systemic lupus erythematosus	0.625444	Focal adhesion	0.501669	Necroptosis	0.525488
Osteoclast differentiation	0.615345	Huntington's disease	0.517828	NOD-like receptor signaling pathway	0.500948	Phagosome	0.513988

## Data Availability

The data used to support the findings of this study are available from the corresponding author upon request.
